# Surgical treatment of infective endocarditis with massive vegetations in a patient with a ventricular septal defect and atopic dermatitis: a case report

**DOI:** 10.1186/s44215-023-00056-z

**Published:** 2023-07-05

**Authors:** Yuta Kitagata, Hiroshi Tsuneyoshi, Kenta Ito, Ken Yamanaka, Masahiro Hirano, Keiichi Hirose, Akio Ikai

**Affiliations:** 1 Department of Cardiovascular Surgery, Shizuoka General Hospital, 4-27-1 Kita Ando Aoi-Ku, Shizuoka City, 420-8527 Japan; 2 Kitano Hospital, Osaka Prefecture, 2-4-20 Ougimachi Kita-Ku, Osaka City, 530-8480 Japan; 3 Department of Clinical Laboratory Medicine, Shizuoka General Hospital, 4-27-1 Kita Ando Aoi-Ku, Shizuoka City, 420-8527 Japan; 4 Department of Adult Congenital Heart Disease, Shizuoka General Hospital, 4-27-1 Kita Ando Aoi-Ku, Shizuoka City, 420-8527 Japan; 5 Department of Cardiovascular Surgery, Mt. Fuji Shizuoka Children’s Hospital, 860 Urushiyama Aoi-Ku, Shizuoka City, 420-8660 Japan

**Keywords:** Infective endocarditis, Ventricular septal defect, Pulmonary artery obstruction, Lung abscess, Atopic dermatitis

## Abstract

**Background:**

It is well known that congenital heart disease, especially a ventricular septal defect, is associated with a high risk of infective endocarditis. There are few reports of infective endocarditis with vegetations extending from the right ventricle into the pulmonary artery, resulting in pulmonary artery embolism. It is also well known that atopic dermatitis can be associated with systemic infections such as infective carditis. Here, we report a patient with a ventricular septal defect and infective endocarditis caused by atopic dermatitis who presented with massively infected vegetations occluding the pulmonary artery and extending from the right ventricle into the pulmonary artery and was treated surgically.

**Case presentation:**

A 26-year-old woman with a ventricular septal defect and a history of atopic dermatitis was diagnosed with infective endocarditis with mobile vegetations in the right ventricle, pulmonary artery occlusion caused by massive vegetations, and pulmonary abscesses. Because the obstructing vegetations did not regress with antibiotics, they were removed surgically and the ventricular septal defect was closed. A new causative organism was identified in the vegetation, enabling optimization of the antibiotic regimen. Appropriate antibiotics were administered for 2 months after surgery, resulting in complete resolution of the lung abscesses.

**Conclusion:**

Aggressive surgical intervention can be effective in patients with massive vegetations obstructing their pulmonary arteries.

## Background

Infective endocarditis (IE) is potentially fatal, and congenital heart disease, especially a ventricular septal defect (VSD), is an important risk factor for it [1]. It is also well known that atopic dermatitis can be associated with systemic infections such as infective carditis [2–4]. There are few reports of IE with vegetations extending from the right ventricle (RV) into the pulmonary artery (PA), resulting in occlusion of that artery. It can be difficult to decide how to treat IE and when to implement treatment. Here, we report our experience of treating a patient with a VSD and IE caused by atopic dermatitis who presented with massively infected vegetations occluding the PA and extending from the RV into the PA.

## Case presentation

**Fig. 1:**
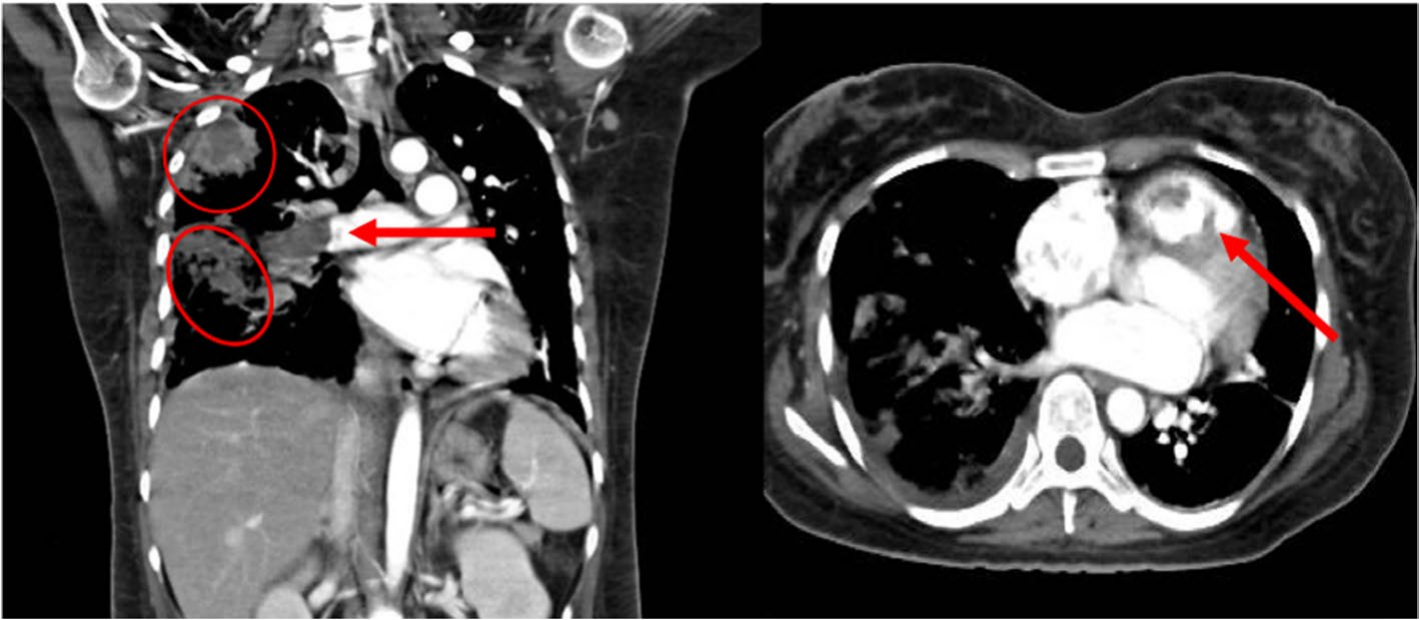
Preoperative enhanced computed tomography on admission showing vegetations in the right ventricle and right PA and abscesses in the right lung

**Fig. 2:**
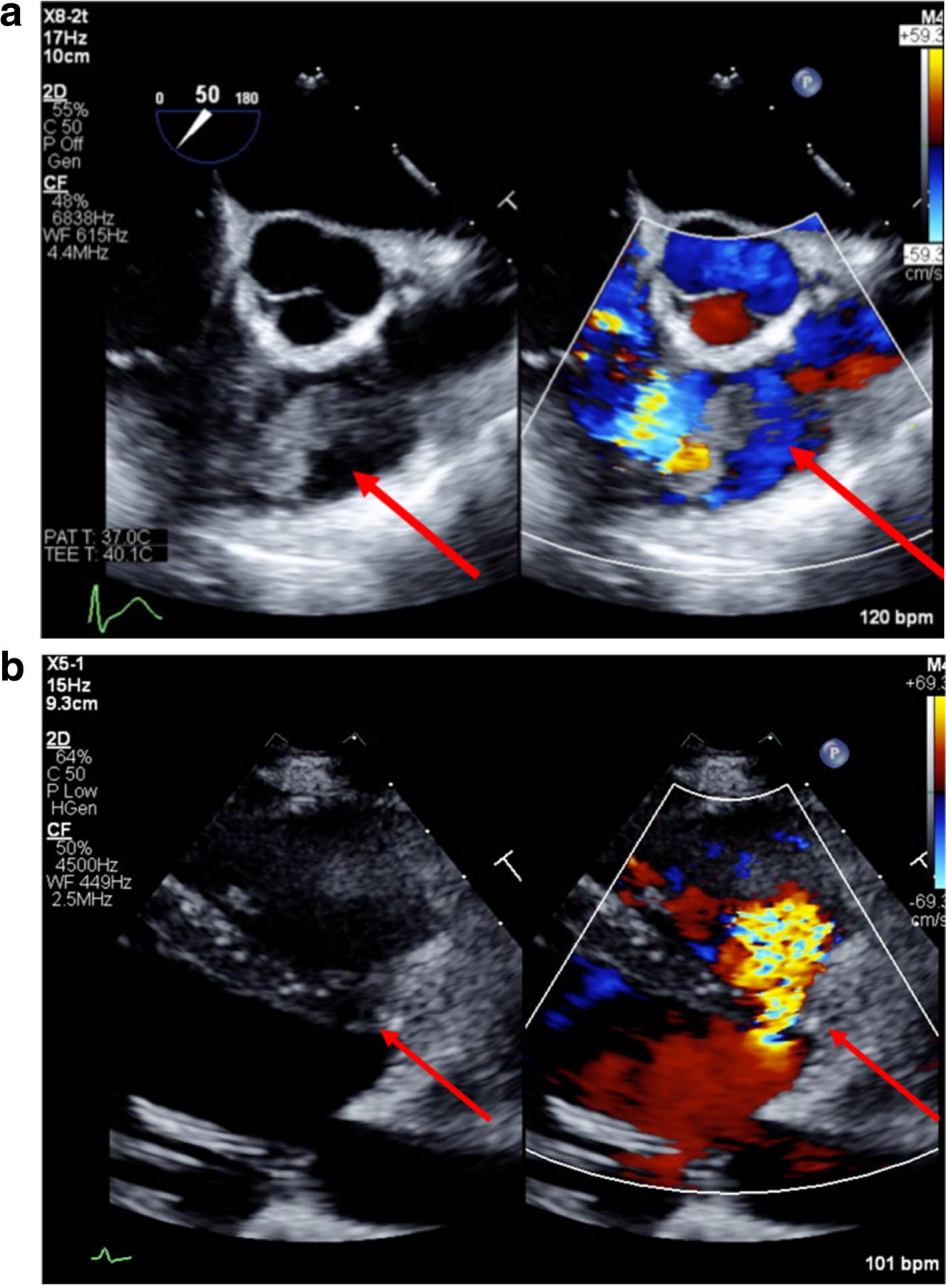
Preoperative transesophageal echocardiography. **a** The vegetation in the right ventricle is located close to the pulmonary valve. **b** The ventricular septal defect jet is directed to the right ventricular wall

A 26-year-old woman was referred to our hospital with dyspnea and high fever after being treated for 2 weeks for IE and septic pulmonary embolism. The vegetation was in the right ventricle and *Staphylococcus aureus* (MSSA) was detected by the blood culture. The patient had atopic dermatitis and a congenital VSD. On examination, she had tachycardia of 130 beats/min, respiratory rate of 25 breaths/min, and oxygen saturation of 98% on 1 L of oxygen. A grade 3/6 pan-systolic murmur was heard at the inferior margin of the left sternum and coarse crackles were noted in both lung fields. Atopic dermatitis and evidence of self-excoriations on the extremities in response to pruritus were noted. Enhanced computed tomography (CT) showed vegetations in the RV and right PA and multiple abscesses in the right lung (Fig. 1). Magnetic resonance imaging of the head showed two asymptomatic cerebral embolisms, which are small infarctions of approximately 1 mm with minute hemorrhage in the dorsal side of the midbrain and medulla oblongata. Echocardiography showed a vegetation in the RV close to the pulmonary valve, with a 5-mm-diameter perimembranous VSD (Fig. 2b, c). MSSA was detected in blood cultures and methicillin-resistant *Staphylococcus epidermidis* (MRSE) on sputum culture. We treated her with clindamycin and cefepime with the intent of improving her hemodynamics and controlling her infection. The choice of antibiotics was considered for meningeal migration and to prevent toxic shock syndrome.

On the basis of her persistent high fever, tachycardia over 120 beats/min, low blood pressure 84/46 mmHg, and lack of response in C-reactive protein concentrations despite antibiotic administration for 5 days, we diagnosed IE refractory to medical therapy with a risk of serious hemodynamic instability. We therefore decided to remove the RV and PA vegetations and close the VSD.

**Fig. 3:**
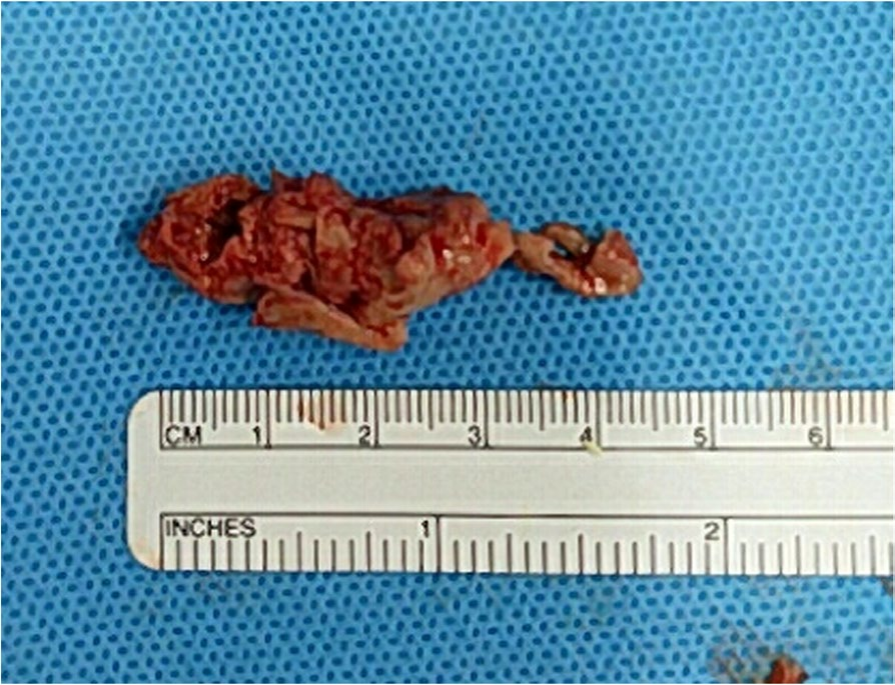
Photograph of the 4.5-cm vegetation that was excised from the right pulmonary artery

After standard initiation of a cardiopulmonary bypass and an incision of right atrium, the VSD was confirmed to be in a perimembranous position behind the anteroseptal commissure of the tricuspid valve. The tricuspid valve was free from the infection. Because of the blood flow through the VSD, fibrous tissue and vegetations were attached to its perimeter. We concluded that the VSD was the cause of the IE. We closed the VSD with a glutaraldehyde-treated autologous pericardial patch. The main PA was then incised, enabling visualization of the pulmonary valve. Because the vegetation was attached to the valve leaflets, the non-facing cusp, including the cusp leaflet, was partially resected and the non-facing cusp was repaired with fresh autologous pericardium. Under moderate hypothermia with low bypass flow, a vertical incision was made in the right PA, revealing that the vegetation continued into the middle and lower lobe branches. The part of the vegetation that was visible was removed (Fig. 3) and the patient was weaned off bypass without difficulty. The cardiopulmonary bypass time was 244 min and the aortic cross-clamp time 147 min.

After intensive respiratory management with inhaled nitric oxide, the patient was extubated on the third postoperative day. Because the MRSE from the excised PA vegetation were resistant to clindamycin, we added vancomycin to the patient’s antibiotic regimen. Postoperative echocardiography showed no remaining vegetations and a small leak in the repaired VSD in the RV. The results of the pathological study for the vegetations were consistent with bacterial IE. After ongoing treatment with antibiotics, enhanced CT on the 6th postoperative day showed that the pulmonary abscesses and unexcised vegetation of the PA had decreased significantly in size. On CT before discharge after ongoing treatment with antibiotics for 2 months, the lesions had almost disappeared completely. The patient was discharged on postoperative day 53 to continue on oral sulfamethoxazole trimethoprim and treatment for dermatitis with oral bepotastine besilate and some ointments. There was no recurrence of infection during 2 years of follow-up.

## Discussion and conclusions

Septic pulmonary embolism can be fatal. In the present case, IE developed in association with adult congenital heart disease. VSD is an important risk factor for IE in adults with congenital heart diseases [1]. Although the European Society of Cardiology guidelines state that surgical treatment should be considered for treatment-resistant IE with embolism associated with congenital heart disease [5], there are no known reports describing this approach [6]. However, some reports describe patients who were treated medically [7, 8].

In our case, we considered that surgical intervention was indicated for three reasons. First, timely removal of the vegetation which made pulmonary circulation worse stabilized our patient’s hemodynamics. She had had ongoing tachycardia and low blood pressure for 5 days, threatening further disruption of her hemodynamics. The surgical procedure achieved stabilization of her hemodynamics, eliminating that risk.

Second, the surgical procedure achieved infection control, as shown by the success of her postoperative antibiotic treatment, in that the pulmonary abscesses resolved. The restricted visual field prevented removal of all of the vegetation in the right PA. However, the improvement in blood flow in the PA and bronchial arteries caused by increased forward flow from the RV enabled sufficient access of antibiotics to the lung tissue. Moreover, MRSE was detected by culture of the PA vegetation obtained surgically. We had administered clindamycin and cefepime preoperatively to target MSSA. However, culture of the surgically obtained sample of vegetation yielded important information regarding the sensitivities of the infecting organism, enabling prescription of more appropriate antibiotics. In this case, MSSA in the blood culture was a true causative organism and the detection of MRSE suggested a localized infection in the lung, so it was not detected in the blood culture. One possibility is that MRSE was detected as the cause of the lung abscesses because MRSE was detected in the PA vegetation and sputum.

Third, VSD closure reduced the risk of recurrence of IE [9] cases, in which self-inflicted trauma related to atopic dermatitis caused systemic infections via blood-borne MSSA [2, 3] and VSD with atopic dermatitis associated with right-sided IE [4] have been reported. Thus, the combination of VSD and atopic dermatitis increases the risk of systemic infection, especially IE. Especially in this case, it was necessary to close the VSD with autologous pericardial patch but artificial patch, not to recurrent IE. In patients with asymptomatic VSD and atopic dermatitis, VSD closure may be indicated to minimize the risk of IE, otherwise ongoing medical support is essential. Regardless of the presence or absence of IE, care must be taken in dealing with asymptomatic patients with VSDs associated with atopic dermatitis. Aggressive surgical intervention may be indicated in some cases.

## Data Availability

All relevant data are in this report.

## Abbreviations

CT: Computed tomography
IE: Infective endocarditis
MRSE: Methicillin-resistant *Staphylococcus epidermidis*
MSSA: Methicillin-susceptible *Staphylococcus aureus*
PA: Pulmonary artery
RV: Right ventricle
VSD: Ventricular septal defect
